# Climate change and the population collapse during the “Great Famine” in pre-industrial Europe

**DOI:** 10.1002/ece3.936

**Published:** 2014-01-02

**Authors:** Mauricio Lima

**Affiliations:** 1 Department of Ecology, Pontificia Universidad Católica de ChileCasilla 114-D, Santiago, CP 6513677, Chile; 2 Laboratorio Internacional de Cambio Global, LINCG (CSIC-PUC)Santiago, CP 6513677, Chile

**Keywords:** Climate change, ecology, human dynamics, population collapse

## Abstract

Population dynamics, economy, and human demography started with Malthus, the idea that population growth is limited by resources and “positive checks” occur when population growth overshoots the available resources. In fact, historical evidence indicates that long-term climate changes have destabilized civilizations and caused population collapses via food shortages, diseases, and wars. One of the worst population collapses of human societies occurred during the early fourteenth century in northern Europe; the “Great Famine” was the consequence of the dramatic effects of climate deterioration on human population growth. Thus, part of my motivation was to demonstrate that simple theoretical-based models can be helpful in understanding the causes of population change in preindustrial societies. Here, the results suggest that a logistic model with temperature as a “lateral” perturbation effect is the key element for explaining the population collapse exhibited by the European population during the “Great Famine”.

## Introduction

The modern sciences of economics, human demography, and population dynamics started with Malthus (1798), who developed the idea that populations grow geometrically, while food resources only expand at an arithmetic rate. The first mathematical model of a population growing in a limited environment was developed by Verhulst (1838) through his logistic equation. This model was used by Pearl and Read (1920) to predict human population growth in the USA, but it failed at predicting population growth at that time. In consequence, demographers abandoned the study of human population size and turned to the study of population age structure and age-specific vital rates. By contrast, population ecologists developed a theory based on simple models and few principles and the statistical analysis of population size changes (Royama 1992; Berryman 1999; Turchin 2003; Ginzburg and Colyvan 2004). After two centuries since the publication of Malthus's book, demographers and population ecologists have started to share a common conceptual framework (Lee 1987; Turchin 2009; Lima and Berryman 2011).

Recent studies have come to recognize that climate has been an important force, shaping the rise and fall of past civilizations (deMenocal 2001; Buckely et al. 2010; Kennett et al. 2012). For example, preindustrial societies experienced an increasing frequency of wars, famines, and epidemics during the periods of climatic deterioration and high population sizes (Zhang et al. 2007, 2011), and there is quantitative evidence of the link between climate and human population growth in preindustrial societies (Büngten et al. 2011; McMichael 2012). Moreover, due the potential threats of global warming, there is a growing focus not only on the historical effects of climate change on human systems (McMichael 2012), but also on the potential role of the present global warming on economy, political stability, and civil war frequency (Hsiang et al. 2011; Scheffran et al. 2012).

One of the worst population collapses of human societies occurred during the early fourteenth century in northern Europe; the “Great Famine” was the consequence of the dramatic effects of climate deterioration on human population growth (Jordan 1996). During this period, the European population collapsed due to the prolonged famine caused by the climatic cooling that was occurring during the transition from the Medieval Warm Period (MWP) to the Little Ice Age (LIA) (Zhang et al. 2007, 2011). The underlying explanation appears to rely on the combination of Malthusian theory (Malthus 1798) with the role of climatic variability as an exogenous forcing factor. Climatic variability determines agricultural land carrying capacity, which in turn affects the population growth of preindustrial societies (Zhang et al. 2007, 2011). However, there is no proper theoretical dynamic modeling for this hypothesis, and no study to date has considered the possibility of climate acting as a “lateral perturbation” (Royama 1992) in human populations. Lateral perturbations are the result of exogenous factors like climate acting on the population equilibrium (or carrying capacity; Royama 1992) and causing nonadditive effects. As equilibrium population sizes are usually set by a resource in short supply (food production or crop yield), it is possible to anticipate lateral perturbation effects whenever climatic variability is suspected of influencing the supply of food. Hence, explanations of climatic deterioration on human population growth during the “Great Famine” need to consider the possible effects of climate on agricultural land carrying capacity, that is, lateral perturbation effects. These nonadditive effects are normally expected when the ratio (i.e., population/crop production) characterizes the per capita share of the resources and the competition strength, changing the availability on a limiting factor. In this scenario, small changes in a climate factor could have large changes in population growth rates because there is an interaction between climate and population size (Royama 1992; Berryman and Lima 2006; Lima et al. 2006).

Thus, part of my motivation was to demonstrate that simple population dynamic models can be helpful in understanding the causes of population change in preindustrial societies. Here, I apply simple models for deciphering the interaction between temperature variability and the dynamics of the European population during the period (800–1800).

## Methods

### Population data

Population data from different regions of Europe during the 800–1600 AD period were extracted from McEvedy and Jones (1978). This source of data about historical human population sizes provides figures for the population of each region/country through historical time. The following regions of western Europe were selected; British Islands, Scandinavian region, France, Germany, Belgium, the Netherlands, Spain, Italy, and all of western Europe. The *R*-function represents the relationship between the per capita growth rate over a given period of time and the human population at the beginning of that period of growth, and can be estimated as:


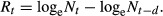
(1)

**Figure 1:**
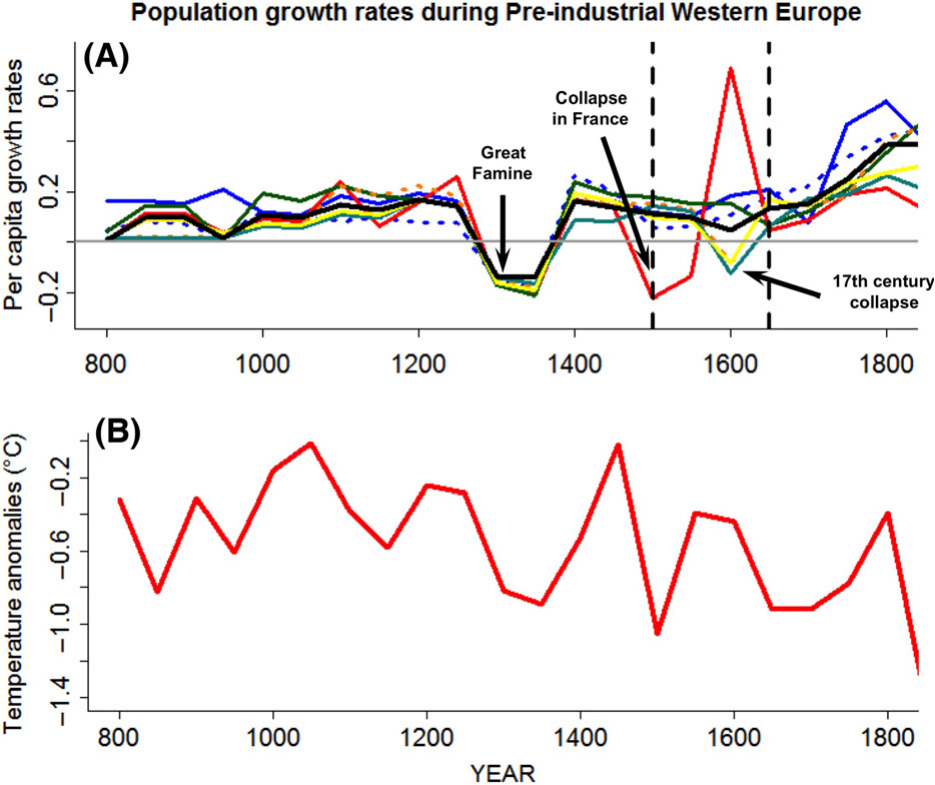
(A) Human population dynamics in preindustrial Europe (AD 800–1800), the time series of the per capita population growth rates log_e_ (*N*_*t*_*/N*_*t−*50y_) are showed for different regions of Europe. Western Europe (Russia excluded; black solid line); British Islands (blue solid line); Scandinavian region (blue dotted line); France (red solid line); Belgium and the Netherlands (green solid line); Germany (orange dotted line); Spain (cyan solid line) and Italy (yellow solid line). Vertical dotted lines showed the time periods used for model fitting (800–1800; 800–1650 and 800–1550). (B) Reconstructed June-July-August temperature anomalies (respect to the 1901–2000 period) time series of 50-years averaged annual values.

*R* can also be expressed in terms of birth and death rates (6):


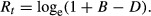
(2)

where *R* is the realized per capita growth rate, *B* and *D* are the per capita birth and mortality rates, respectively, and *d* is the time period between population estimates. In this study, the time scale for the population analyses was 50 years (Fig. 1A). Because McEvedy and Jones' (1978) population size data are at irregular time intervals, the natural logarithm of the data points was taken, linearly interpolated and then anti-logged back, to create time series with an interval of 50 years (Fig. S1). Although the generational time step for humans is around 30–25 years (Turchin 2009), I preferred to use an interval of 50 years in order to reduce the number of interpolated values in the database.

### Climatic data

To describe the historical temperature variability in Europe for the period (800–1800), the Central Europe 2500 Year Tree Ring Summer Climate Reconstructions was used (Büngten et al. 2011). The climatic data set is a tree ring-based reconstruction of central European summer temperatures over the past 2500 years. The original annual temperature data set was averaged on 50 years' time steps to make the temperature time series compatible with the time step used for the human population time series (Fig. 1B). I prefer to use this reconstructed temperature data because is based in one proxy (tree rings), and it considers a large time period. Others temperature reconstructed data are multiproxy (tree rings, documentaries, pollen assemblages, and ice cores) adding an extra source of variation in the reconstructed data (Guiot et al. 2010).

### Theoretical models of human population dynamics

The starting point was to model human dynamics using the exponential growth model that is a fundamental property of all population systems (Berryman 1999), I used the discrete time version:


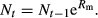
(3)

By defining equation 3 in terms of the *R*-function using equation 1 and log transforming the equation 3, a model of exponential growth is obtained, where the additive effects of climate can be added:


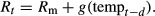
(4)

where *R*_*t*_ is the realized per capita growth rate estimated from data (*R*_*t*_* = *log_e_ *N*_*t*_ *–* log_e_ *N*_*t−*1_), *R*_m_ is a positive constant representing the maximum per capita growth rate, and *g* is a simple linear function (+ or −) of the temperature anomalies at different lags. Model 4 represents the dynamics of a human population in a varying unlimited environment.

The alternative hypothesis is that human population was limited by resources (food, land, crop yield). I therefore used a simple model of intra-specific competition, the generalized exponential form of the discrete logistic model (Royama 1992; Berryman 1999):


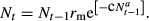
(5)

where *N*_*t*_ represents the population size at time *t*,*r*_m_ is a positive constant representing the maximum finite reproductive rate, *c* is a constant representing competition and resource depletion, and *a* indicates the effect of interference on each individual as population size increases (Berryman 1999). A value of *a *> 1 indicates that competition intensifies with population size, and *a* < 1 indicates that per capita competition decreases with population size. The parameter *c* represents the equilibrium population size (Berryman 1999). By defining equation 5 in terms of the *R*-function, defining *R*_*t*_* = *log_e_ *N*_*t*_ *–* log_e_ *N*_*t−*1_, log-transforming equation 5, and defining the population size in natural logarithm *X*_*t*_* *= log_e_ (*N*_*t*_), the Ricker (1954) model of equation 5 can be expressed as:


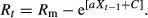
(6)

where *R*_*t*_ is the realized per capita growth rate *R*_*t*_ = log_e_ (*N*_*t*_*/N*_*t*−1_), *R*_m_* *= log_e_(*r*_m_), *a* is the same parameter as in equation 5, *C* = log_e_(c), and *X *= log_e_(*N*). Therefore, this model can be used for representing the hypothesis that temperature variability has an impact on food supplies through the effect on land-carrying capacity in preindustrial societies (McMichael 2012). The correct model in this scenario is that the carrying capacity (equilibrium point) is affected by climate. In this case, the climatic factor (temperature) shifts the *R*-function curve along the *x*-axis without changing the slope at equilibrium, representing a “lateral” perturbation in the Royama (1992) framework:


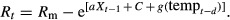
(7)

Models from equations 4 and 7 were fitted by nonlinear least squares using the *nls* library in the R program (R Development Core Team 2011, available at http://www.r-project.org) and ranked according to the Bayesian information criterion (BIC) or Schwarz criterion (Schwarz 1978). For clarity, BIC weights were also included in the results. Minimum BIC was selected to determine the best model. Finally, simulations were carried out to elucidate the capacity of the models to describe the real dynamics. Simulations were carried out only using the first real value of the time series, and then, running the algorithm using each model with their estimated parameters to obtain the simulated values. I used the Pearson's correlation coefficient between the observed and predicted numbers to assess model predictions.

## Results

Human population growth rates in Europe during the AD 800–1800 period were characterized by irregular fluctuations and positive values interrupted by some important population collapses (Fig. 1A). In particular, the widely reported crisis of the fourteenth century, the population collapse observed in France during the period 1500–1600, and a smaller population collapse recorded during the first half of the seventeenth century in some regions (Fig. 1A). The per capita growth rates showed the magnitude of the human collapse experienced by the European population during the fourteenth century (Fig. 1A). After the second half of the seventeenth century all regions of western Europe started a strong acceleration in the per capita growth rates (Fig. 1A).

According to the modeling analyses for the period 800–1800, a simple exponential model with the exogenous direct effects of temperature accounts for 2% and 15% of the observed variation in *R* values of the European population across the different regions (Table S1). The addition of the reconstructed lagged temperatures increased the explained variance to 3% and 16% in the different regions (Table S1). The BIC values indicate that the simplest exponential models including the positive direct temperatures effect were the selected models (Table S1). However, the BIC weights were not able to give strong empirical support for any of these models. In fact, no model was able to explain more than 25% of the variance in population growth rates (Table S1).

**Figure 2:**
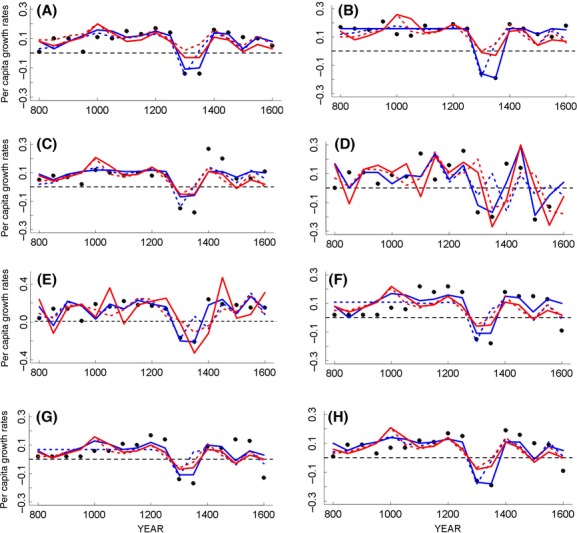
Comparison of observed human per capita population growth rates (solid dots) for the period AD 800–1650 with predictions from the models fitted to the data (Table 1). Red lines are the predictions of exponential population growth models with additive direct effects of temperature (dotted lines) and additive direct and lagged effects of temperatures (solid lines). Blue lines are the predictions of logistic population growth models with non-additive (lateral) effects of direct temperatures (dotted lines) and nonadditive (lateral) effects of direct and lagged temperatures (solid lines). (A) Western Europe; (B) British Islands; (C) Scandinavian region; (D) France; (E) Belgium and the Netherlands; (F) Germany; (G) Spain and (H) Italy.

The next step was to split the time series for the period 800–1650, and the modeling analyses showed that simple exponential models with the exogenous direct effects of temperature accounts for 2% and 38% of the observed variation in *R* values of the European population across the different regions (Table 1). The addition of the reconstructed lagged temperatures increased the explained variance to 15% and 44% in the different regions (Table 1). However, the BIC values and the BIC weights of the logistic models, including direct and lagged temperatures as lateral perturbations, indicate strong empirical support for these models in almost all cases (Table 1). For three regions, France, Germany, and Spain, the logistic models including direct and lagged temperatures showed relatively lower explained variance than other regions (Table 1; 46%, 54%, and 46%, respectively) and smaller ΔBIC values compared with the other models (Table 1). However, these simple logistic models with the direct and lagged temperatures as lateral perturbation effects were quite accurate in predicting the population collapse observed during the 14th century as well as the posterior recovery and even some of the other collapses observed during the sixteenth and seventeenth centuries (Fig. 2). Model predictions of population growth rates in France failed to simulate the population collapses and recoveries (Fig. 2). All regions but France showed positive lateral effects of direct (50 years) and lagged (100 years) summer temperatures (Table 1), France was the only region that showed negative direct effects of temperature (Table 1). However, I fitted the same models for the period 800–1550, most of the regions showed very similar parameter values (Table S1), but France, Germany, and Spain regions showed an important increase in the explained variance (France from 46% to 62%; Germany from 54% to 70%, and Spain from 48% to 66%). Moreover, the best models in these regions showed positive lateral effects of direct and lagged temperatures and were able to predict quite well the population collapses and recoveries (Fig. S2).

**Table 1:** Population dynamic models for the preindustrial European Population (800–1650 AD) using a pure exponential model with additive effects of temperature and the exponential form of logistic growth with lateral effects of temperature (Royama 1992); parameter values are given in the equations. The best model was chosen by using the Bayesian Information Critera (BIC).

Population models (period 800–1650)	Log-likelihood	BIC	*P*	ΔBIC	*w*_*i*_	*r*^2^	*r* (predictions)
Europe							
* R*_*t*+1_ = 0.220 + 0.290 Temp_*t*_	20.70	*−*32.89	3	6.94	0.006	0.36	0.61
* R*_*t*+1_ = 0.267 + 0.271 Temp_*t*_ + 0.120 Temp_*t−*50_	21.57	*−*31.81	4	8.02	0.006	0.42	0.66
* R*_*t*+1_ = 0.146 −exp[* − *0.812 *X*_*t*_ − 2.77−5.88 Temp_*t*_]	23.97	*−*33.77	5	6.06	0.022	0.56	0.76
* R*_*t*+1_ = 0.170 −exp[* − *0.62 *X*_*t*_ − 3.38 − 4.35 Temp_*t*_ − 1.97 Temp_*t−*50_]	27.00	*−*39.83	5	0.00	0.962	0.71	0.85
British Islands							
* R*_*t*+1_ = 0.281 + 0.328 Temp_*t*_	15.90	*−*23.31	3	32.02	0.0001	0.29	0.54
* R*_*t*+1_ = 0.366 + 0.291 Temp_*t*_ + 0.219 Temp_*t−*50_	17.60	*−*23.86	4	31.47	0.0001	0.43	0.66
* R*_*t*+1_ = 0.179 −exp[*−*0.117 *X*_*t*_ − 6.82 − 6.90 Temp_*t*_]	19.48	*−*24.80	5	30.53	0.0001	0.53	0.73
* R*_*t*+1_ = 0.162 −exp[0.550 *X*_*t*_ − 18.91 − 14.24 Temp_*t*_ − 8.23 Temp_*t−*50_]	36.17	*−*55.33	6	0.000	1.000	0.94	0.97
Scandinavian Region							
* R*_*t*+1_ = 0.231 + 0.331 Temp_*t*_	18.30	*−*28.10	3	6.09	0.013	0.36	0.58
* R*_*t*+1_ = 0.289 + 0.306 Temp_*t*_ + 0.142 Temp_*t−*50_	19.30	*−*27.27	4	6.92	0.013	0.43	0.66
* R*_*t*+1_ = 0.149 −exp[* − *0.935 *X*_*t*_ − 5.29−5.41 Temp_*t*_]	20.77	*−*27.36	5	6.83	0.021	0.52	0.73
* R*_*t*+1_ = 0.117 −exp[* − *1.56 *X*_*t*_ − 9.72 − 8.24 Temp_*t*_ − 3.90 Temp_*t−*50_]	25.59	*−*34.19	6	0.00	0.952	0.73	0.83
France							
* R*_*t*+1_ = 0.189 + 0.210 Temp_*t*_	2.78	2.94	3	1.26	0.095	0.04	*−*0.23
* R*_*t*+1_ = 0.342 + 0.144 Temp_*t*_ + 0.395 Temp_*t−*50_	3.92	3.50	4	1.82	0.108	0.15	0.14
* R*_*t*+1_ = 0.158 −exp[11.45 *X*_*t*_ − 29.00 + 4.28 Temp_*t*_]	5.41	3.34	5	1.66	0.178	0.29	*−*0.09
* R*_*t*+1_ = 0.169 −exp[27.47 *X*_*t*_ − 69.83 + 13.58 Temp_*t*_ − 7.95 Temp_*t−*50_]	7.66	1.68	6	0.00	0.621	0.46	0.47
Belgium and the Netherlands							
* R*_*t*+1_ = 0.305 + 0.385 Temp_*t*_	14.72	*−*20.93	3	11.27	0.024	0.33	0.46
* R*_*t*+1_ = 0.376 + 0.355 Temp_*t*_ + 0.181 Temp_*t−*50_	15.68	*−*20.02	4	12.18	0.042	0.40	0.40
* R*_*t*+1_ = 0.203 − exp[* − *0.630 *X*_*t*_ − 5.80−6.12 Temp_*t*_]	17.38	*−*20.59	5	11.61	0.015	0.52	0.42
* R*_*t*+1_ = 0.200 − exp[* − *0.916 *X*_*t*_ − 9.11 − 7.60 Temp_*t*_ − 3.90 Temp_*t−*50_]	23.18	*−*32.20	5	0.00	0.917	0.77	0.76
Germany							
* R*_*t*+1_ = 0.242 + 0.343 Temp_*t*_	14.72	*−*20.93	3	1.74	0.26	0.28	0.54
* R*_*t*+1_ = 0.294 + 0.321 Temp_*t*_ + 0.135 Temp_*t−*50_	15.23	*−*19.14	4	3.53	0.10	0.32	0.57
* R*_*t*+1_ = 0.107 − exp[3.29 *X*_*t*_ − 15.27 − 7.75 Temp_*t*_]	15.47	*−*16.77	5	5.90	0.03	0.34	0.52
* R*_*t*+1_ = 0.180 − exp[* − *0.856 *X*_*t*_ − 4.39 − 4.57 Temp_*t*_ − 2.12 Temp_*t−*50_]	18.42	*−*22.67	5	0.00	0.61	0.54	0.76
Spain							
* R*_*t*+1_ = 0.182 + 0.285 Temp_*t*_	18.33	*−*28.16	3	0.00	0.38	0.29	0.53
* R*_*t*+1_ = 0.229 + 0.265 Temp_*t*_ + 0.120 Temp_*t−*50_	18.97	*−*26.60	4	1.56	0.17	0.34	0.59
* R*_*t*+1_ = 0.07 − exp[8.18 *X*_*t*_ − 23.65 − 6.56 Temp_*t*_]	20.31	*−*26.46	5	1.70	0.16	0.44	0.48
* R*_*t*+1_ = 0.160 − exp[* − *0.444 *X*_*t*_ − 3.69 − 2.91 Temp_*t*_ − 1.42 Temp_*t−*50_]	20.86	*−*27.56	5	0.60	0.28	0.48	0.73
Italy							
* R*_*t*+1_ = 0.239 + 0.361 Temp_*t*_	17.85	*−*27.22	3	6.67	0.03	0.38	0.61
* R*_*t*+1_ = 0.288 + 0.340 Temp_*t*_ + 0.129 Temp_*t−*50_	18.56	*−*25.78	4	8.11	0.02	0.44	0.64
* R*_*t*+1_ = 0.162 − exp[* − *0.291 *X*_*t*_ − 4.12−4.31 Temp_*t*_]	20.03	*−*25.89	5	8.00	0.02	0.52	0.72
* R*_*t*+1_ = 0.140 − exp[* − *0.232 *X*_*t*_ − 6.302 − 4.88 Temp_*t*_ − 2.57 Temp_*t−*50_]	25.44	*−*33.89	6	0.00	0.93	0.75	0.81

*R*_*t+*1_, Realized per capita growth rates; *X_t_*, ln population size; Temp_*t*_, Mean reconstructed temperatures during the 50-year interval; Temp_*t−*50_, Mean reconstructed temperatures during the lagged 50-year interval; *Δ*BIC, model BIC – lowest BIC; *w*_*i*_, BIC weigths; *r*^*2*^, proportion of the variance explained by the model; *r* predictions, Pearson's correlation coefficient between the observed and predicted dynamics.

## Discussion

Several recent studies have revealed the statistical relationship between climate change, war frequencies and population declines in agrarian societies (Zhang et al. 2007, 2011; McMichael 2012). The postulated mechanism for explaining the observed population collapses in Europe is the direct link between long-term climate and land carrying capacity (Zhang et al. 2007). The simple logistic model used in this study represents a strong conceptual support for the role of Malthusian forces operating in preindustrial societies, in particular until the seventeenth century. Despite social scientists having been discussing the role of Malthusian factors in shaping human population dynamics (Lee 1987; Lee and Anderson 2002), no previous study has attempted to model environmental fluctuations (climate) as a lateral effect that directly affects the limiting factors in the long term (food production). However, recent studies combining climatic reconstructions, food production, population growth rates, and war frequency data have provided empirical support for the logistic models (Zhang et al. 2007, 2011; Lee et al. 2008, 2009).

A simple logistic Ricker model, where the carrying capacity is a function of the long-term temperature average, appears to describe the dynamics of the pre-industrial human population in Europe quite well. In fact, the positive relationship between per capita growth rates and human population sizes observed in some regions of Europe during the 800–1200 period can be explained as a positive lateral effect caused by an increasing agricultural productivity due to the medieval warm period (MWP). In a previous study, Lima and Berryman (2011) suggested that cooperation could be the engine of change during this period. Nevertheless, this study offers a simpler explanation, and the fitted logistic models were able to explain the increasing trend (800–1200 BC) and the great population collapse at the beginning of the fourteenth century. This collapse can be interpreted as the consequence of nonadditive long-term effects of temperature on human population dynamics. Temperature represents an exogenous factor influencing land availability and productivity (food production), which determined the population size at equilibrium in European agrarian societies. It is interesting to note that recent studies explain the population collapse recorded during the fourteenth century as a consequence of climate cooling associated with the onset of the Little Ice Age (LIA), causing a severe decline in per capita food production and subsequent famines, wars and spread of the black pest (Zhang et al. 2007, 2011). In fact, previous studies have hypothesized that “food supply per capita” is the key variable driving population collapses in pre-industrial societies (Zhang et al. 2011), the key element for capturing this variable is the interaction between the long-term temperature trend and human population size. When climate affects the availability of a particular limiting factor (e.g., food production), the per capita resource share of individuals is also influenced (Royama 1992). As a consequence, the effect of the climatic variable cannot be evaluated independently of the population size, because the exogenous effect (temperature) acts in conjunction with population size (Royama 1992; Berryman and Lima 2006; Lima et al. 2006), resulting in potentially nonlinear responses of populations to changes in climate. Therefore, the population collapse suffered by the human population in Europe during the “Great Famine” was caused by the interaction between climate cooling and the high population size that had resulted from previous centuries of warm climatic conditions.

It is important to note the strong nonlinearity detected in all logistic models of human dynamics (*a* coefficient in equations 5^7^) suggesting that the effect of individual competition or interference may become stronger as population size increases. The combination of nonlinearity and exogenous lateral perturbation effects is the ideal condition to produce population responses disproportionate to the magnitude or intensity of the exogenous forcing effect.

An interesting result of this study is that by the middle of the seventeenth century almost all regions of western Europe began to exhibit acceleration in per capita population growth rates that ended by the middle of the eighteenth century. This suggests that in western Europe the rapid increase in population growth rates triggered a century before the industrial revolution. In fact, logistic models were unable to capture the variability in growth rates when the analysis is extended to the eighteenth century. This positive feedback in population growth rates supports the hypothesis of the fundamental role of human population size for triggering technological innovation, increased food production and economic development (Boserup 1965, 1981). This idea is in agreement with the earlier ecological concept of cooperative interactions and aggregations accentuating the growth of animal populations (Allee 1932; Lima and Berryman 2011).

Finally, this article illustrates how general ecological theory can be employed to understand and explain the causes of human population collapses in preindustrial societies. The dynamics of the preindustrial European population and the “Great Famine” collapse can be explained by the logistic theory and climate change. In some manner, it is a reconciliation of logistic model and human dynamics after the controversial article by Pearl and Read (1920). This is an example of applied ecological theory, in particular, the application of the logistic equation and theories pertaining to nonlinear population dynamics and exogenous perturbations for dealing with demographic changes in human societies.

## Supporting Information

Figure S1 Human population dynamics in pre-industrial Western Europe (AD 800–1800); the time series of population size estimated from McEvedy and Jones (1978) are showed (blue closed dots) together with the interpolated data at 50 year time step intervals (red closed dots; see material and methods). a) Western Europe (Russia excluded; b) British Islands; c) Scandinavian region; d) France; e) Belgium and Netherlands; f) Germany; g) Spain and h) Italy.

Figure S2 Comparison of observed human per capita population growth rates (solid dots) for the period AD 800–1550 with predictions from the models fitted to the data (Table S1). Blue lines are the predictions of logistic population growth models with non-additive (lateral) effects of direct temperatures (dotted lines) and non-additive (lateral) effects of direct and lagged temperatures (solid lines); a) France; b) Germany and c) Spain.

Table S1 Population dynamic models for the preindustrial European Population (800–1800 AD/800–1550 AD) using a pure exponential model with additive effects of temperature and the exponential form of logistic growth with lateral effects of temperature (Royama 1992); parameter values are given in the equations.
