# Supplementary figures and images for: Climate change and the population collapse during the “Great Famine” in pre-industrial Europe
Source: Ecol Evol. 2014 Jan 2;4(3):284–91. doi: 10.1002/ece3.936 (PMC3925430; doi:10.1002/ece3.936)

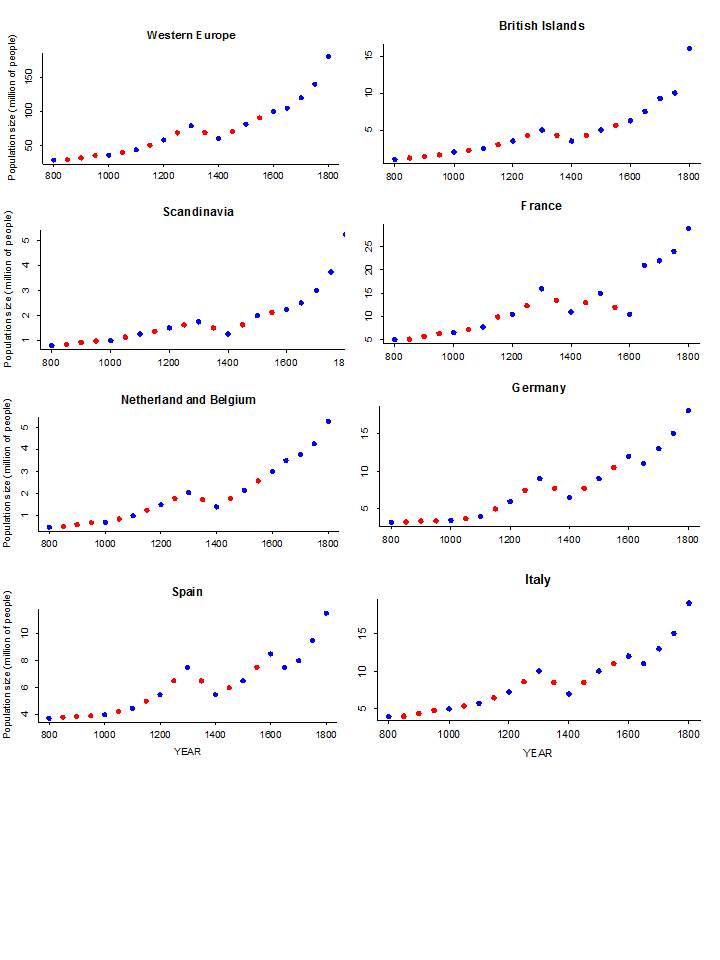

Supplement: Figure S1 — Human population dynamics in pre-industrial Western Europe (AD 800–1800); the time series of population size estimated from McEvedy and Jones (1978) are showed (blue closed dots) together with the interpolated data at 50 year time step intervals (red closed dots; see material and methods). a) Western Europe (Russia excluded; b) British Islands; c) Scandinavian region; d) France; e) Belgium and Netherlands; f) Germany; g) Spain and h) Italy. [file ece30004-0284-sd1.jpg]

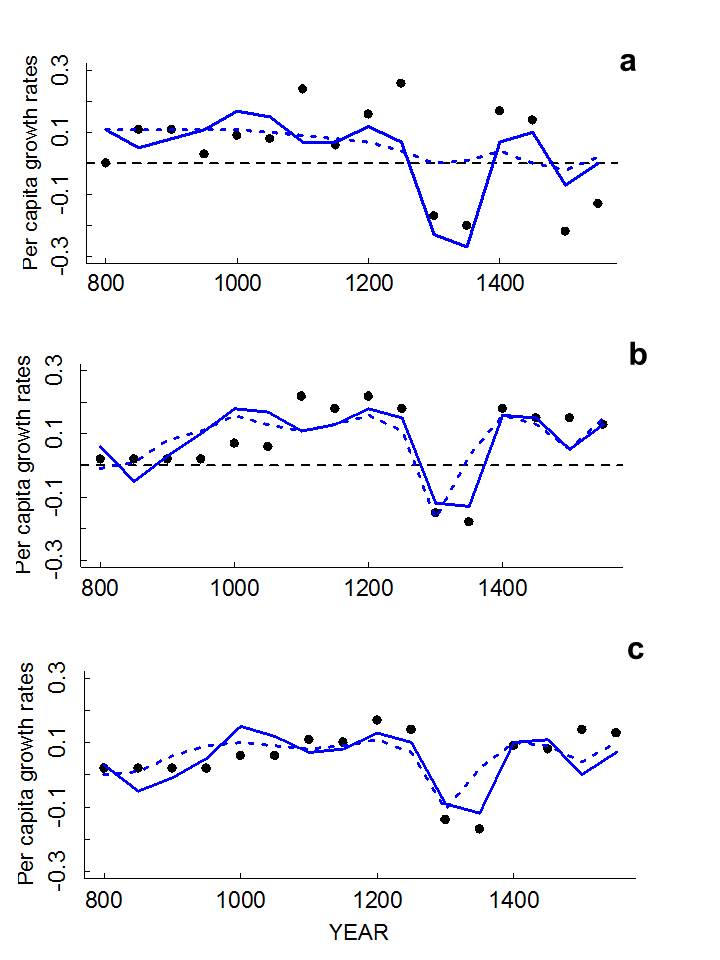

Supplement: Figure S2 — Comparison of observed human per capita population growth rates (solid dots) for the period AD 800–1550 with predictions from the models fitted to the data (Table S1). Blue lines are the predictions of logistic population growth models with non-additive (lateral) effects of direct temperatures (dotted lines) and non-additive (lateral) effects of direct and lagged temperatures (solid lines); a) France; b) Germany and c) Spain. [file ece30004-0284-sd2.jpg]
