# Supplementary material for: Climate change and the population collapse during the “Great Famine” in pre-industrial Europe
Source: Ecol Evol. 2014 Jan 2;4(3):284–91. doi: 10.1002/ece3.936 (PMC3925430; doi:10.1002/ece3.936)
Supplement: Table S1 — Population dynamic models for the preindustrial European Population (800–1800 AD/800–1550 AD) using a pure exponential model with additive effects of temperature and the exponential form of logistic growth with lateral effects of temperature (Royama 1992); parameter values are given in the equations. [file ece30004-0284-sd3.docx]

**Table S1**. Population dynamic models for the pre-industrial European Population (800-1800 AD/800-1550 AD) using a pure exponential model with additive effects of temperature and the exponential form of logistic growth with lateral effects of temperature (Royama 1992); parameter values are given in the equations. The best model was chosen by using the Bayesian Information Critera (BIC). *R _t+1_* = Realized per capita growth rates, *X _t+1_* = ln population size, *Temp _t_* = Mean reconstructed temperatures during the 50-year interval, *Temp _t-50_* = Mean reconstructed temperatures during the lagged 50-year interval, *∆BIC* = model BIC – lowest BIC, *w_i_* = BIC weigths, *r^2^* = proportion of the variance explained by the model.

| **Population models (period 800-1800)** | **Log-likelihood** | **BIC** | ***p*** | **∆BIC** | ***w_i_*** | **r^2^** |
| --- | --- | --- | --- | --- | --- | --- |
| **Europe** |  |  |  |  |  |  |
| 1. **R_t+1_ = 0.124 + 0.062 Temp_t_** | **19.62** | **-30.26** | **3** | **0.00** | **0.49** | **0.04** |
| 1. R_t+1_ = 0.098 + 0.065 Temp_t_ - 0.055 Temp_t-50_ | 19.98 | -27.98 | 4 | 2.28 | 0.16 | 0.08 |
| 1. R_t+1_ = 0.300 - exp[-0.284 X_t_ - 0.674 - 0.149 Temp1_t_] | 20.60 | -29.23 | 4 | 1.03 | 0.29 | 0.13 |
| 1. R_t+1_ = 0.300 - exp[-0.266 X_t_ - 0.708 - 0.476 Temp_t_ + 0.062 Temp_t-50_] | 20.62 | -26.27 | 5 | 3.99 | 0.07 | 0.13 |
| **British Islands** |  |  |  |  |  |  |
| 1. **R_t+1_ = 0.179 + 0.076 Temp_t_** | **12.66** | **-16.33** | **3** | **0.00** | **0.64** | **0.03** |
| 1. R_t+1_ = 0.167 + 0.078 Temp_t_ - 0.025 Temp_t-50_ | 12.69 | -13.40 | 4 | 2.93 | 0.15 | 0.04 |
| 1. R_t+1_ = 0.500 - exp[-0.064 X_t_ - 1.09 - 0.287 Temp_t_] | 12.82 | -13.66 | 4 | 2.67 | 0.17 | 0.05 |
| 1. R_t+1_ = 0.500 - exp[-0.063 X_t_ - 1.09 - 0.286 Temp_t_ + 0.006 Temp_t-50_] | 12.82 | -10.66 | 5 | 5.67 | 0.04 | 0.05 |
| **Scandinavian Region** |  |  |  |  |  |  |
| 1. R_t+1_ = 0.121 + 0.052 Temp_t_ | 14.68 | -20.37 | 3 | 0.92 | 0.28 | 0.02 |
| 1. R_t+1_ = 0.068 + 0.060 Temp_t_ - 0.115 Temp_t-50_ | 15.66 | -19.35 | 4 | 2.02 | 0.16 | 0.11 |
| 1. **R_t+1_ = 0.300 - exp[-0.540 X_t_ - 1.71 - 0.64 Temp_t_]** | **16.64** | **-21.29** | **4** | **0.00** | **0.44** | **0.19** |
| 1. R_t+1_ = 0.300 - exp[-0.467 X_t_ - 1.57 - 0.57 Temp_t_ + 0.27 Temp_t-50_] | 16.87 | -18.76 | 5 | 2.53 | 0.12 | 0.21 |
| **France** |  |  |  |  |  |  |
| 1. **R_t+1_ = 0.216 + 0.244 Temp_t_** | **5.99** | **-2.99** | **3** | **0.00** | **0.69** | **0.15** |
| 1. R_t+1_ = 0.225 + 0.242 Temp_t_ + 0.019 Temp_t-50_ | 6.00 | -0.02 | 4 | 2.97 | 0.16 | 0.15 |
| 1. R_t+1_ = 0.164 - exp[-0.276 X_t_ - 5.48 – 5.01 Temp_t_] | 7.25 | 0.48 | 5 | 3.47 | 0.12 | 0.25 |
| 1. R_t+1_ = 0.163 - exp[-0.314 X_t_ - 5.62 - 5.22 Temp_t_ - 0.092 Temp_t-50_] | 7.25 | 3.47 | 6 | 6.46 | 0.03 | 0.25 |
| **Belgium and Netherlands** |  |  |  |  |  |  |
| 1. **R_t+1_ = 0.196 + 0.149 Temp_t_** | **16.18** | **-23.37** | **3** | **0.00** | **0.64** | **0.15** |
| 1. R_t+1_ = 0.187 + 0.150 Temp_t_ - 0.023 Temp_t-50_ | 16.21 | -20.44 | 4 | 2.93 | 0.15 | 0.16 |
| 1. R_t+1_ = 0.220 - exp[-0.206 X_t_ - 3.06 - 1.50 Temp_t_] | 16.30 | -20.62 | 4 | 2.75 | 0.16 | 0.17 |
| 1. R_t+1_ = 0.220 - exp[-0.350 X_t_ - 3.62 - 1.95 Temp_t_ - 0.59 Temp_t-50_] | 16.59 | -18.19 | 5 | 5.18 | 0.05 | 0.19 |
| **Germany** |  |  |  |  |  |  |
| 1. **R_t+1_ = 0.121 + 0.068 Temp_t_** | **15.10** | **-21.22** | **3** | **0.00** | **0.71** | **0.03** |
| 1. R_t+1_ = 0.116 + 0.069 Temp_t_ - 0.012 Temp_t-50_ | 15.12 | -18.25 | 4 | 2.97 | 0.16 | 0.03 |
| 1. R_t+1_ = 0.169 - exp[-0.613 X_t_ - 2.08 - 1.26 Temp_t_] | 15.55 | -16.11 | 5 | 5.11 | 0.05 | 0.07 |
| 1. R_t+1_ = 0.180 - exp[-0.945 X_t_ - 2.14 - 1.73 Temp_t_ - 0.91 Temp_t-50_] | 15.90 | -16.82 | 5 | 4.40 | 0.08 | 0.11 |
| **Spain** |  |  |  |  |  |  |
| 1. **R_t+1_ = 0.076 + 0.039 Temp_t_** | **17.91** | **-26.83** | **3** | **0.00** | **0.72** | **0.02** |
| 1. R_t+1_ = 0.056 + 0.041 Temp_t_ - 0.043 Temp_t-50_ | 18.09 | -24.20 | 4 | 2.63 | 0.19 | 0.03 |
| 1. R_t+1_ = 0.123 - exp[-0.786 X_t_ - 1.81 - 0.83 Temp_t_] | 18.09 | -21.19 | 5 | 5.64 | 0.04 | 0.03 |
| 1. R_t+1_ = 0.189 - exp[-0.330 X_t_ - 1.69 - 0.42 Temp_t_ + 0.10 Temp_t-50_] | 18.09 | -21.21 | 5 | 5.62 | 0.04 | 0.03 |
| **Italy** |  |  |  |  |  |  |
| 1. **R_t+1_ = 0.111 + 0.064 Temp_t_** | **16.14** | **-23.29** | **3** | **0.00** | **0.60** | **0.03** |
| 1. R_t+1_ = 0.082 + 0.068 Temp_t_ - 0.063 Temp_t-50_ | 16.46 | -20.94 | 4 | 2.35 | 0.19 | 0.06 |
| 1. R_t+1_ = 0.220 - exp[-0.294 X_t_ - 1.69 - 0.606 Temp_t_] | 16.36 | -20.74 | 4 | 2.55 | 0.17 | 0.05 |
| 1. R_t+1_ = 0.220 - exp[-0.199 X_t_ - 1.72 - 0.523 Temp_t_ + 0.240 Temp_t-50_] | 16.45 | -17.91 | 5 | 5.38 | 0.04 | 0.06 |
| **Population models (period 800-1550)** | **Log-likelihood** | **BIC** | ***p*** | **∆BIC** | ***w_i_*** | **r^2^** |
| **Europe** |  |  |  |  |  |  |
| 1. R_t+1_ = 0.222 + 0.299 Temp_t_ | 17.41 | -26.69 | 3 | 9.01 | 0.01 | 0.36 |
| 1. R_t+1_ = 0.272 + 0.269 Temp_t_ + 0.142 Temp_t-50_ | 18.33 | -25.84 | 4 | 8.86 | 0.01 | 0.44 |
| 1. R_t+1_ = 0.150 - exp[-0.919 X_t_ - 2.46 - 6.08 Temp1_t_] | 20.35 | -27.16 | 5 | 7.54 | 0.02 | 0.56 |
| 1. **R_t+1_ = 0.113 - exp[-3.26 X_t_ - 6.40 - 17.24 Temp_t_ - 7.83 Temp_t-50_]** | **25.47** | **-34.70** | **6** | **0.00** | **0.96** | **0.77** |
| **British Islands** |  |  |  |  |  |  |
| 1. R_t+1_ = 0.298 + 0.376 Temp_t_ | 14.06 | -20.00 | 3 | 32.83 | 0.00 | 0.36 |
| 1. R_t+1_ = 0.376 + 0.329 Temp_t_ + 0.221 Temp_t-50_ | 15.56 | -20.28 | 4 | 32.55 | 0.00 | 0.48 |
| 1. R_t+1_ = 0.198 - exp[0.248 X_t_ - 5.57 - 4.85 Temp_t_] | 17.70 | -21.86 | 5 | 30.97 | 0.00 | 0.61 |
| 1. **R_t+1_ = 0.170 - exp[-0.072 X_t_ - 15.66 - 12.50 Temp_t_ - 6.95 Temp_t-50_]** | **33.19** | **-52.83** | **5** | **0.00** | **1.00** | **0.95** |
| **Scandinavian Region** |  |  |  |  |  |  |
| 1. R_t+1_ = 0.242 + 0.368 Temp_t_ | 17.57 | -26.84 | 3 | 3.82 | 0.11 | 0.42 |
| 1. R_t+1_ = 0.295 + 0.342 Temp_t_ + 0.138 Temp_t-50_ | 18.46 | -25.82 | 4 | 4.84 | 0.07 | 0.47 |
| 1. R_t+1_ = 0.160 - exp[-0.605 X_t_ - 4.70 - 4.55 Temp_t_] | 19.40 | -24.94 | 5 | 5.72 | 0.04 | 0.55 |
| 1. **R_t+1_ = 0.117 - exp[-1.37 X_t_ - 9.34 - 7.78 Temp_t_ - 3.74 Temp_t-50_]** | **23.65** | **-30.66** | **6** | **0.00** | **0.77** | **0.73** |
| **France** |  |  |  |  |  |  |
| 1. R_t+1_ = 0.263 + 0.447 Temp_t_ | 10.87 | -13.43 | 3 | 5.29 | 0.06 | 0.32 |
| 1. R_t+1_ = 0.384 + 0.389 Temp_t_ + 0.318 Temp_t-50_ | 13.06 | -15.04 | 4 | 3.68 | 0.13 | 0.48 |
| 1. R_t+1_ = 0.112 - exp[4.95 X_t_ - 15.49 - 0.856 Temp_t_] | 12.29 | -10.72 | 5 | 8.00 | 0.01 | 0.43 |
| 1. **R_t+1_ = 0.160 - exp[0.966 X_t_ - 6.82 – 2.22 Temp_t_ - 2.27 Temp_t-50_]** | **15.29** | **-18.72** | **5** | **0.00** | **0.80** | **0.62** |
| **Belgium and Netherlands** |  |  |  |  |  |  |
| 1. R_t+1_ = 0.318 + 0.426 Temp_t_ | 14.04 | -19.75 | 3 | 12.41 | 0.00 | 0.38 |
| 1. R_t+1_ = 0.383 + 0.394 Temp_t_ + 0.169 Temp_t-50_ | 14.88 | -18.67 | 4 | 13.49 | 0.00 | 0.44 |
| 1. R_t+1_ = 0.211 - exp[-0.453 X_t_ - 5.20 - 5.30 Temp_t_] | 16.09 | -18.31 | 5 | 13.85 | 0.00 | 0.52 |
| 1. **R_t+1_ = 0.170 - exp[-2.87 X_t_ - 21.45 - 19.88 Temp_t_ - 9.43 Temp_t-50_]** | **23.01** | **-32.16** | **5** | **0.00** | **1.00** | **0.81** |
| **Germany** |  |  |  |  |  |  |
| 1. R_t+1_ = 0.231 + 0.309 Temp_t_ | 13.84 | -19.36 | 3 | 8.60 | 0.01 | 0.24 |
| 1. R_t+1_ = 0.287 + 0.280 Temp_t_ + 0.147 Temp_t-50_ | 14.46 | -17.83 | 4 | 10.09 | 0.01 | 0.29 |
| 1. R_t+1_ = 0.165 - exp[-1.36 X_t_ - 3.99 - 6.77 Temp_t_] | 16.72 | -19.57 | 5 | 8.35 | 0.01 | 0.46 |
| 1. **R_t+1_ = 0.170 - exp[-1.98 X_t_ - 5.45 - 7.74Temp_t_ - 3.44 Temp_t-50_]** | **20.89** | **-27.92** | **5** | **0.00** | **0.97** | **0.70** |
| **Spain** |  |  |  |  |  |  |
| 1. R_t+1_ = 0.169 + 0.244 Temp_t_ | 17.83 | -27.35 | 3 | 6.18 | 0.04 | 0.24 |
| 1. R_t+1_ = 0.221 + 0.218 Temp_t_ + 0.135 Temp_t-50_ | 18.71 | -26.32 | 4 | 7.21 | 0.02 | 0.32 |
| 1. R_t+1_ = 0.109 - exp[-1.94 X_t_ - 3.72 - 7.17 Temp_t_] | 20.05 | -26.23 | 5 | 7.30 | 0.02 | 0.42 |
| 1. **R_t+1_ = 0.140 - exp[-1.90 X_t_ - 3.68 - 5.26 Temp_t_ - 2.67 Temp_t-50_]** | **23.69** | **-33.53** | **5** | **0.00** | **0.91** | **0.66** |
| **Italy** |  |  |  |  |  |  |
| 1. R_t+1_ = 0.230 + 0.333 Temp_t_ | 16.80 | -25.28 | 3 | 11.81 | 0.00 | 0.35 |
| 1. R_t+1_ = 0.283 + 0.306 Temp_t_ + 0.140 Temp_t-50_ | 17.62 | -24.15 | 4 | 12.94 | 0.00 | 0.41 |
| 1. R_t+1_ = 0.145 - exp[-0.94 X_t_ - 4.24 - 6.11 Temp_t_] | 19.70 | -25.53 | 5 | 11.56 | 0.00 | 0.55 |
| 1. **R_t+1_ = 0.140 - exp[-1.47 X_t_ - 6.57 - 7.75 Temp_t_ - 3.66 Temp_t-50_]** | **24.48** | **-37.09** | **5** | **0.00** | **0.99** | **0.79** |

**Figure captions**

**Figure S1.** Human population dynamics in pre-industrial Western Europe (AD 800-1800); the time series of population size estimated from McEvedy and Jones (1978) are showed (blue closed dots) together with the interpolated data at 50 year time step intervals (red closed dots; see material and methods). a) Western Europe (Russia excluded; b) British Islands; c) Scandinavian region; d) France; e) Belgium and Netherlands; f) Germany; g) Spain and h) Italy.

**Figure S2.** Comparison of observed human per capita population growth rates (solid dots) for the period AD 800-1550 with predictions from the models fitted to the data (Table S1). Blue lines are the predictions of logistic population growth models with non-additive (lateral) effects of direct temperatures (dotted lines) and non-additive (lateral) effects of direct and lagged temperatures (solid lines); a) France; b) Germany and c) Spain.

**Literature cited**

McEvedy C, Jones R (1978) *Atlas of world population history*. Allen Lane, London, UK
